# A core extended naphtalene diimide G-quadruplex ligand potently inhibits herpes simplex virus 1 replication

**DOI:** 10.1038/s41598-017-02667-3

**Published:** 2017-05-24

**Authors:** Sara Callegaro, Rosalba Perrone, Matteo Scalabrin, Filippo Doria, Giorgio Palù, Sara N. Richter

**Affiliations:** 1 Department of Molecular Medicine, University of Padua, via Gabelli 63, 35121 Padua, Italy; 2 Department of Chemistry, University of Pavia, V.le Taramelli 10, 27100 Pavia, Italy

## Abstract

G-quadruplexes (G4s) are nucleic acids secondary structures, epigenetic regulators in cells and viruses. In herpes simplex virus 1 (HSV-1)-infected cells, G4s are massively present during viral replication. We here aimed at investigating the possibility to target the HSV-1 G4s by a core extended naphtalene diimide (c-exNDI) G4 ligand. Biophysical and biomolecular analysis proved that c-exNDI stabilized the HSV-1 G4s in a concentration dependent manner. In MS competition assays, c-exNDI preferentially recognized HSV-1 G4s over cellular telomeric G4s, the most represented G4s within cells; other less abundant cellular G4s were also recognized. Treatment of HSV-1 infected cells with c-exNDI at low nanomolar concentrations induced significant virus inhibition with no cytotoxicity. The mechanism of action was ascribed to G4-mediated inhibition of viral DNA replication, with consequent impairment of viral genes transcription. Our data suggest that the observed potent antiviral activity and low cytotoxicity mainly depend on a combination of c-exNDI affinity for HSV-1 G4s and their massive presence during infection. HSV-1 G4s may thus represent new effective antiviral targets: the fact that no current antiherpetic drug exploits them and their presence at the viral genome, responsible for both active and latent HSV infections, makes them particularly attracting.

## Introduction

G-quadruplexes (G4s) are nucleic acids secondary structures that may form in single-stranded G-rich sequences under physiological conditions^1^. Four Gs bind via Hoogsteen-type hydrogen bonds base-pairing to yield G-quartets, which stack to form the G4. The presence of K^+^ cations specifically supports G4 formation and stability^2^. Based on the strand orientation, G4s can adopt three main topologies: parallel, antiparallel, and hybrid-type structures. In eukaryotes, G4s have been shown to be involved in key regulatory roles, including transcriptional regulation of gene promoters and enhancers, translation, chromatin epigenetic regulation, DNA recombination^3–7^. Expansion of G-quadruplex-forming motifs has been associated with relevant human neurological disorders^4, 8, 9^. Formation of G4s *in vivo* has been consolidated by the discovery of cellular proteins that specifically recognize G4s^10, 11^ and the development of G4 specific antibodies^12, 13^.

Recently, the presence of G4s in viruses and their involvement in virus key steps has been provided^14^. G4s have been implicated in pathogenic mechanisms of the human immunodeficiency virus, where functionally significant G4s have been identified^5, 11, 15–17^ and stabilized by G4 ligands with consequent antiviral effects^5, 18, 19^. G4s have been reported in the SARS coronavirus^20^, the human papilloma, Zika, Ebola and hepatitis C virus genome^21–24^. Among herpesviruses, RNA G4s have been implicated in the regulation of DNA replication and translation of the Epstein–Barr virus^25, 26^. We have shown that the herpes simplex virus 1 (HSV-1) possesses several repeats of important G4-forming sequences, which could be stabilized by a G4 ligand with inhibition of viral DNA replication^27^. In addition, HSV-1 G4s, visualized with the aid of a G4-specific antibody in infected cells^12^, were shown to form massively in the cell nucleus, peak during viral replication and localize according to the viral genome intracellular movements^28^.

The involvement of G4 structures in several human diseases propelled the development of small molecules directed against G4s^7, 29^. However, only very few have been tested against viruses, i.e. BRACO-19 (HIV-1, EBV and HSV-1), pyridostatin (EBV) and Core- Extended Naphtalen Diimide compounds (c-exNDIs) (HIV-1)^5, 18, 19, 25, 26^. Since no definitive drugs have been found against most viral infections, there is an obvious need for new more active compounds.

C-exNDI derivatives have been shown to display fair selectivity towards the HIV-1 G4s that form in the LTR viral promoter versus cellular G4s^19^. This selectivity rests on the preferred recognition of the loop regions of the HIV-1 vs cellular G4s. We here sought to investigate if the best anti-HIV-1 compound of the series, c-exNDI 2 in our previous work^19^, displayed also anti-HSV-1 activity and to test its mechanism of action.

We found that c-exNDI was able to bind and stabilize the HSV-1 G4 forming sequences in a concentration dependent manner. Treatment of HSV-1 infected cells with c-exNDI induced a complete inhibition of the virus at low nanomolar concentration, with a mechanism of action directed toward viral DNA replication. Inhibition of viral DNA replication impaired viral genes transcription resulting in an effective antiherpetic effect. MS competition assays demonstrated that c-exNDI preferentially binds HSV-1 G4s over cellular telomeric G4, the most represented G4s within cells, suggesting that the observed preferential antiviral activity vs cytotoxicity is mediated by both the higher amount of HSV-1 G4s in the cell and their higher affinity for c-exNDI.

## Results

### The G4 ligand c-exNDI highly stabilizes the HSV-1 G4s

We have previously shown that c-exNDIs, compounds that were specifically developed against HIV-1 G4s, were indeed able to selectively recognize and bind with high affinity the HIV-1 G4 sequences^19^. The human telomeric G4-folded sequence and the G4s forming in the promoters of some oncogenes were also bound, albeit with lower affinity. To test the possibility to expand their antiviral activity, we here evaluated the ability of the lead member of the c-exNDIs family, c-exNDI 2 in^19^ (herein called c-exNDI) to interact also with the G4s forming in the terminal repeats of HSV-1.

Three sequences were considered: two sequences forming a four-stacked-G-quartet structure (*un2* and *gp054a*) and one forming a three-G-quartet G4 (*un3*)^27^. Stability and conformation changes upon addition of c-exNDI were detected by circular dichroism (CD).

**Figure 1:**
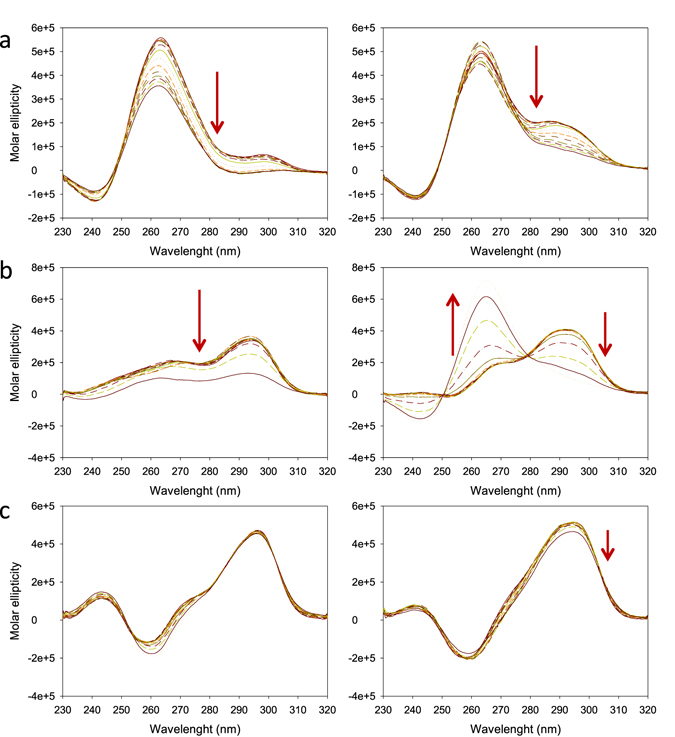
Thermal unfolding of *un3* parallel G4 (**a**), *gp054a* hybrid G4 (**b**), *un2* antiparallel G4 (**c**) (4 μM) in 100 mM K^+^ in the absence (left panel) or presence (right panel) of 16 μM c-exNDI. Thermal unfolding was recorded over a temperature range of 20–90 °C with temperature increase of 5 °C.

In 100 mM K^+^, c-exNDI stabilized *un3* and *gp054a* by >24.9 °C and 15.2 °C, respectively (Table 1), and induced a slight change in the oligonucleotide G4 conformation at 20 °C (Fig. 1a,b). In the case of *gp054a*, during thermal unfolding, the presence of the compound showed an increased stabilization of an alternative parallel-like conformation (Fig. 1b). *Un2* G4 displayed a starting T_m_ above 90 °C in 100, 20 and 5 mM K^+^, therefore no appreciable ΔT_m_ value could be obtained. However, a mild CD spectrum variation of *un2* upon addition of c-exNDI indicated interaction (Fig. 1c): in addition, in the absence of K^+^, an appreciable ΔT_m_ was obtained with c-exNDI (Table 1). These data indicate that c-exNDI is able to effectively bind and stabilize all HSV-1 G4s.

**Table 1:** Melting temperatures (T_m_, °C) of HSV-1 G4 folding sequences measured by CD in the absence or presence (100 mM) of KCl. Each sequence (4 μM) was analyzed in the absence or presence of c-exNDI (16 μM). nd stands for “not detected”.

Melting temperature						
G4 sequence	KCl 0 (°C)		ΔT_m_ (°C)	KCl 100 mM (°C)		ΔT_m_ (°C)
c-exNDI	—	+		—	+	
*gp054a*	nd	nd	—	64.3 ± 0.84	79.5 ± 0.61	15.2
*un2*	50.6 ± 0.53	56.8 ± 0.62	6.2	>90	>90	—
*un3*	nd	nd	—	65.1 ± 3.03	>90	>24.9

**Figure 2:**
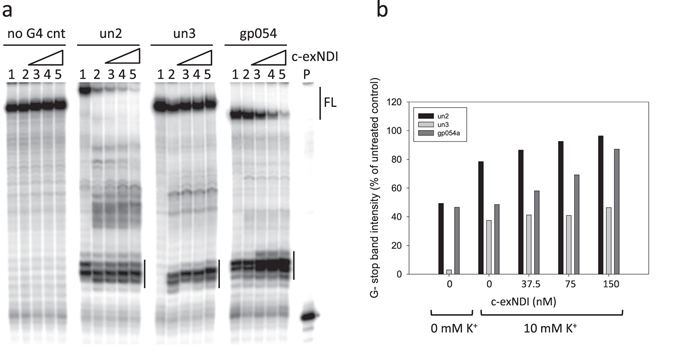
*Taq* polymerase stop assay on HSV-1 G4 folding sequences in the presence of c-exNDI. (**a**) *un2*, *un3* and *gp054a* were analyzed in the absence (lanes 1) or presence of 10 mM K^+^ (lanes 2–5). c-exNDI was used at concentrations of 37.5, 75 and 100 nM (lanes 3–5). Elongation was performed at 60 °C. A non-G4 folding sequence was used as negative control (no G4 cnt). G4-related stops are indicated by vertical bars. FL stands for “full-length” amplified template. P indicates the primer lane. (**b**) Quantification of c-exNDI-induced stop sites observed in (**a**).

To confirm the increased stability of the viral G4s in the presence of the compound, a *Taq* polymerase stop assay was set up. Templates corresponding to *un2*, *un3* and *gp054a* sequences (Table S1) were annealed to a primer and incubated with *Taq* polymerase for 30 min at 60 °C. A sequence unable to fold into G4 was alongside assayed as negative control. In the absence of K^+^, *un2* and *gp054a* displayed a marked stop site corresponding to the first G of the most 3′ G-tract (Fig. 2a, lanes 1 *un2* and *gp054*), indicating stable G4 folding. The stop site increased upon addition of K^+^ and c-exNDI (Fig. 2a, lanes 2–5 *un2* and *gp054*); moreover, in the presence of the compound the full-length amplified product sharply decreased, indicating effective stabilization of *un2* and *gp054a* G4s and thus inhibition of polymerase progression (Fig. 2a, lanes 3–5 *un2* and *gp054*). In the absence of K^+^ no stop site was observed in the *un3* sequence, which thus did not form G4 in these conditions (Fig. 2a, lane 1 *un3*); upon addition of K^+^ and c-exNDI, a marked stop became visible at the most 3′ G-tract (Fig. 2a, lanes 2–5 *un3*), indicating effective stabilization also of this template. Quantification of the stop sites indicated a similar degree of polymerase stalling induced by the compound in the three G4-forming sequences (Fig. 2b).

To check the selectivity of c-exNDI for HSV-1 G4s, mass spectrometry (MS) competition was performed. Selected competitor G4s were hTel, c-myc and c-kit2 G4s, which correspond to the G4-forming sequences of the human telomeric repeat, the promoter of the c-myc oncogene and c-kit protooncogene^30, 31^, and HIV-1 LTR-III G4, the HIV-1 G4 previously shown to be selectively bound with high affinity by c-exNDI^5, 19^. In each case, HIV-1 LTR-III G4s were preferred over HSV-1 G4s, data that confirm c-exNDI selectivity for HIV-1 G4s^19^ (Table 2). C-myc G4 was also always preferred and c-kit2 G4 was preferred *vs* HSV-1 *un2* and *gp054a*. In contrast, HSV-1 *un2* and *un3* G4s were preferred over hTel. *Gp054a* was generally less preferentially bound over all the other analysed sequences. The measured selectivity did not depend on the starting stability of the competing G4s: in fact, in 100 mM K^+^, preferred G4s such as LTR-III (T_m_ 52.9 ± 0.2 °C) and c-myc (T_m_ > 90 °C) had T_m_ values both lower and higher, respectively, than HSV-1 G4s (Table 1); in contrast, non-preferred G4s had similar T_m_ values (hTel21 68.6 ± 0.2, c-kit2 72.9 ± 1.1 °C) (Table 1).

**Table 2:** Relative binding affinity, analyzed by MS competition assay for *un2*, *un3*, *gp054a*, hTel21, c-kit2 (kit), c-myc (myc) and HIV-1 LTR-III G4-folded oligonucleotides.

Binding affinity c-exNDI^19^					
Competing G4s	un2	un3	gp054a	LTR-III	Cell (hTel/myc/kit)
*un2*/LTR-III	20			58	
*un2*/kit	24				46
*un2*/myc	15				68
*un2*/hTel	34				30
*un3*/LTR-III		43		61	
*un3*/kit		52			31
*un3*/myc		36			66
*un3*/hTel		72			21
*gp054a*/LTR-III			3	75	
*gp054a*/kit			7		64
*gp054a*/myc			4		78
*gp054a*/hTel			12		39

### C-exNDI displays potent anti-HSV-1 activity

**Figure 3:**
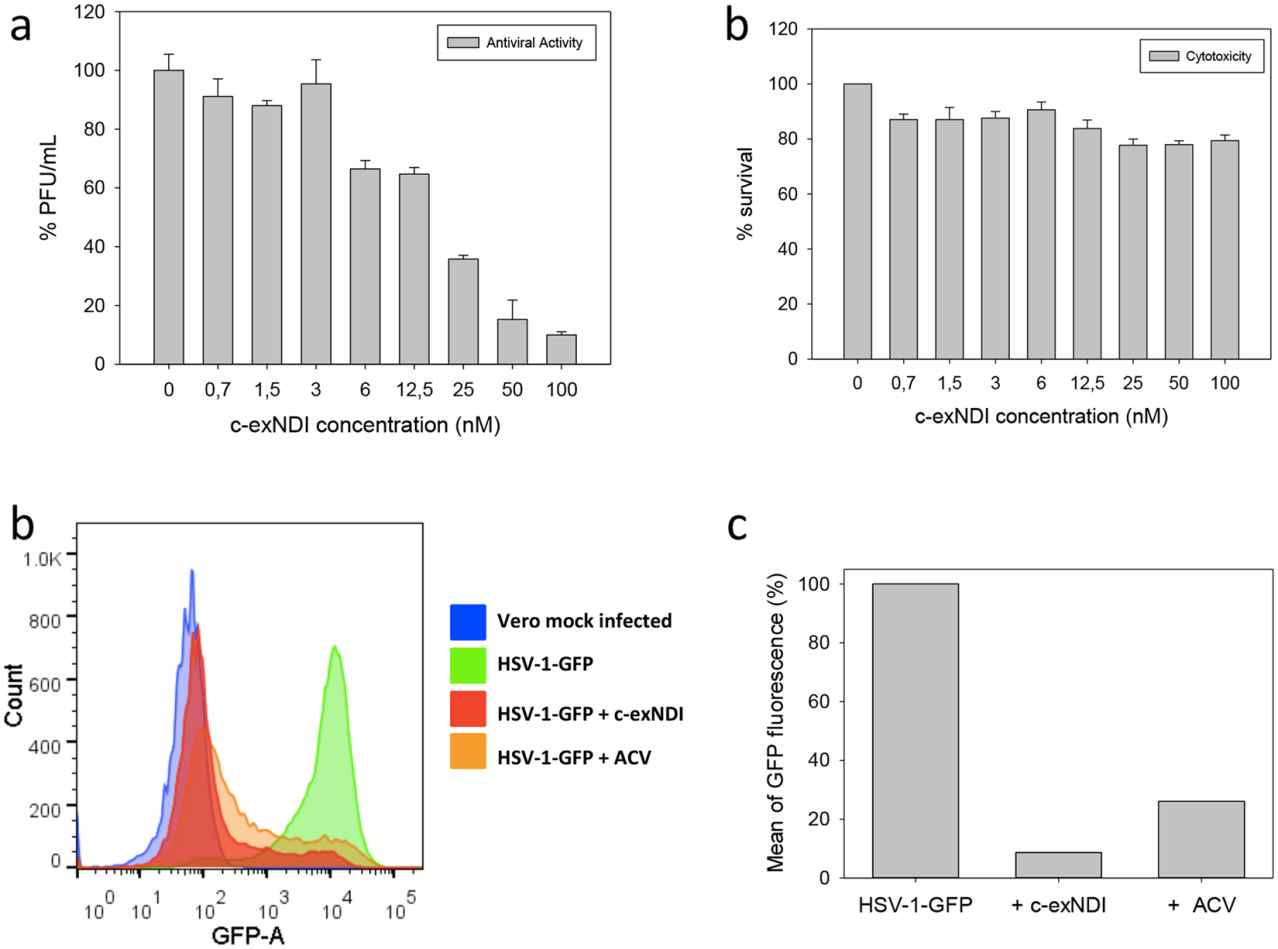
Anti-HSV-1 activity of c-exNDI. (**a**) Plaque assay: Vero cells were infected with HSV-1 strain F (MOI 1, as previously reported^27^) and treated with increasing concentrations of c-exNDI (0.7 nM–100 nM). Supernatants were collected 24 h.p.i. and the number of plaque forming units was determined. (**b**) Vero cells were treated at the same concentrations of c-exNDI (0.7 nM–100 nM) used in the antiviral assay and cytotoxicity was evaluated by MTT assay. (**c**) Flow cytometry of HSV-1 [V41]-infected cells treated with c-exNDI and ACV. HSV-1 [V41]-infected cells were used as positive control and their GFP fluorescence set to 100%. C-exNDI (red curve) and ACV (orange curve)-treated infected cells were compared to HSV-1 [V41]-infected cells (green curve) to monitor compound inhibition of GFP fluorescence. (**d**) Quantification of the mean of GFP fluorescence upon treatment of HSV-1 [V41]-infected cells with c-exNDI and ACV, observed in (*b*).

Since our data indicate that even if HSV-1 G4s are not totally preferred, nonetheless they are preferred over telomeric G4s, the most abundant cellular G4 structures in infected cells, we tested the ability of c-exNDI to inhibit HSV-1. In plaque assay, the compound showed 50% inhibition of HSV-1 production (IC_50_) at 18.3 ± 1.4 nM (Fig. 3a). Interestingly, the compound concentration able to kill 50% of the cells, as measured by MTT assay, was 628.4 ± 2.1 nM, resulting in a remarkable selectivity index (SI = 34.3 ± 2.7) (Fig. 3a). To confirm this data, a recombinant HSV-1 expressing GFP fused to the viral protein VP16 (HSV-1 [V41]) was used to infect cells; this mutant virus is characterized by normal replication kinetics and yields^32^. Analysis was performed by flow cytometry. Cells were treated with c-exNDI and acyclovir (ACV), the antiviral drug of choice for treatment of HSV-1 infections^33^, here used as the reference drug to which compare the anti-HSV-1 potency of c-exNDI. Both compounds were used at low/non cytotoxic concentration (i.e. 100 nM c-exNDI and 3 μM ACV) corresponding to 5 times their IC_50_ values^34, 35^. Cells infected with HSV-1 [V41] and treated with c-exNDI or ACV at 24 h.p.i were monitored for their GFP fluorescence. C-exNDI (Fig. 3b,c) induced an almost complete reduction of GFP fluorescence (8.6%, expressed as mean of GFP fluorescence), whereas 26% of cells remained fluorescent upon treatment with ACV (Fig. 3b,c).

**Figure 4:**
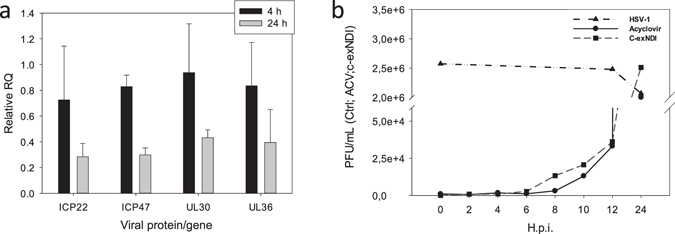
Antiviral effects of c-exNDI. (**a**) Effect of c-exNDI on mRNA levels of immediate-early (IE), early (E) and late (L) proteins of HSV-1. Infected cells were treated with c-exNDI (100 nM); 4 or 24 h.p.i total RNA was isolated, retrotranscribed into cDNA and expression of specific genes was determined by RT-PCR. RQ are Relative Quantities. Each gene was analyzed in duplicate. In all data sets: n ≥ 2, mean ± s.d., Student’s t-test, p ≤ 0.01. (**b**) Effect of c-exNDI on HSV-1 cycle steps evaluated by time of addition assay. C-exNDI was tested at 100 nM (dashed line/squares), acyclovir (ACV) was used as a reference drug and tested at 3 μM (solid line/circles). Data from infected cells treated in the same conditions but without the compounds are reported as dashed line/triangles. Compounds were administered from 0 to 12 h.p.i. and supernatants were collected 30 h.p.i. The left and right Y-axis refer to ACV and c-exNDI data, respectively.

To investigate the mechanism of action of c-exNDI, mRNA levels of representative viral genes were measured at 4 and 24 h.p.i. upon treatment with c-exNDI. In particular, genes corresponding to immediate-early (IE: ICP22, ICP47), early (E: UL30) and late (L: UL36) proteins were considered. At 4 h.p.i., no significant inhibition of viral mRNAs production was observed, whereas at 24 h.p.i. all mRNAs were reduced to a similar extent (28–45%) (Fig. 4a). A similar trend has been reported for ACV^36^. To assess the main and temporally last viral step targeted by c-exNDI, we performed a time of addition (TOA) assay where the maximal low/mild-cytotoxic concentration of the compound was added at different times post-infection (corresponding to different viral cycle steps). The compound maintained its inhibitory activity when added up to 8 h.p.i (Fig. 4b). This is the time when viral replication occurs; indeed, ACV, a known inhibitor of the viral DNA polymerase^37^, showed a remarkably overlapping TOA profile. These data indicate that c-exNDI inhibits viral production mainly by targeting viral replication. Inhibition of viral DNA replication was further confirmed by qPCR analysis. At 4 h.p.i no reduction in viral DNA replication and production was observed, whereas at 24 h.p.i viral DNA was reduced of about 40% (Fig. S1), similarly to what observed with another G4-ligand targeting HSV-1 DNA^27^.

## Discussion

**Figure 5:**
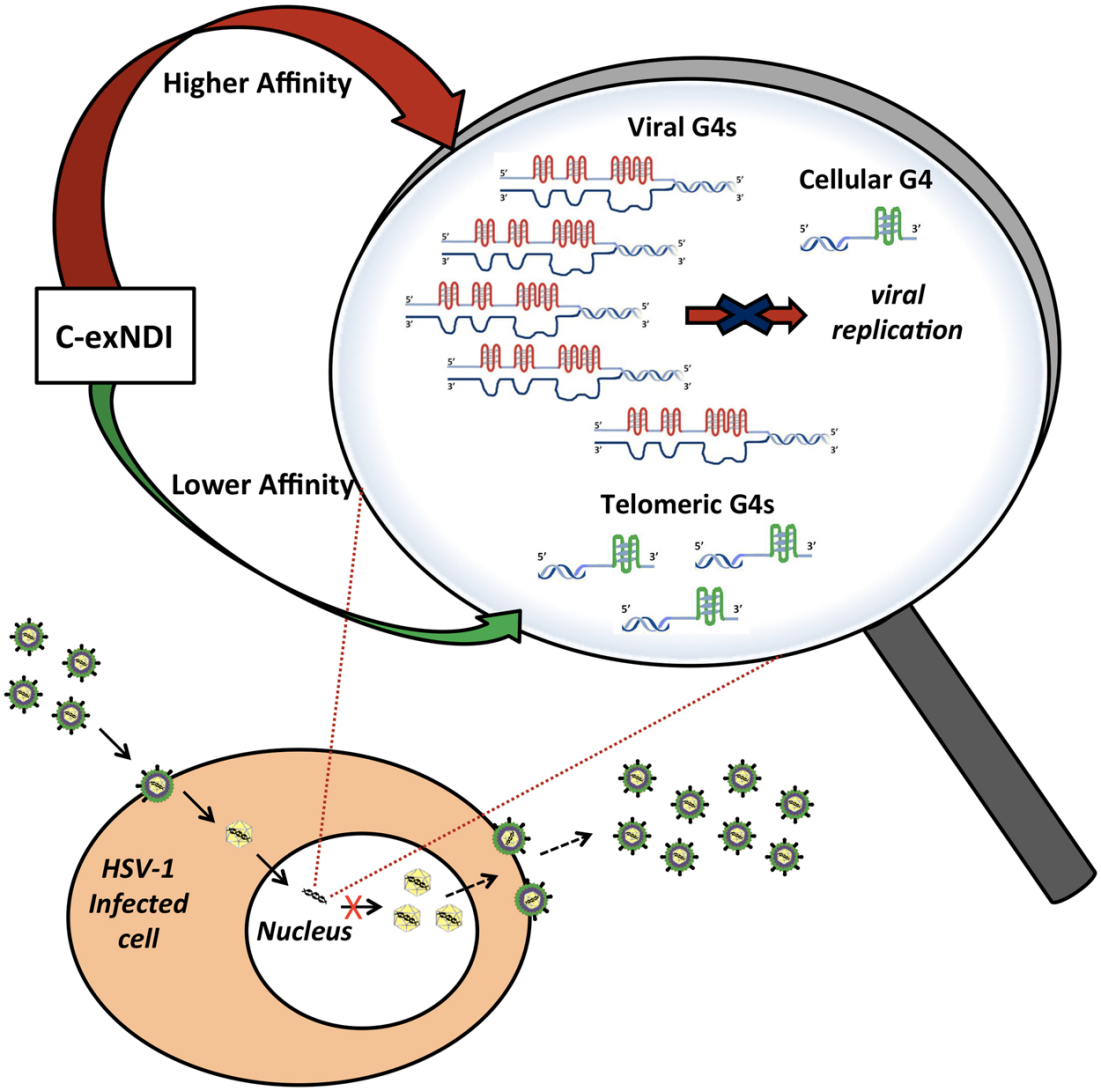
Scheme of the proposed c-exNDI mechanism of anti-HSV-1 activity.

We have shown that c-exNDI, a compound initially developed to specifically interact with HIV-1 G4s, potently stabilizes also G4s found in the HSV-1 genome. Interestingly, even if a net preferentiality for HSV-1 G4s over some cellular G4s was not observed, nevertheless c-exNDI displayed potent anti-HSV-1 activity paralleled by mild/low toxicity at the doses necessary to achieve complete suppression of virus production (Fig. 3a). We ascribe this behaviour to two main reasons: i) the massive presence of G4s during the HSV-1 viral cycle. By an antibody-based approach in cells we demonstrated that viral G4s form massively in the nucleus during viral replication^28^. During this step they likely control both viral replication itself and other key processes of the infection, with consequent dramatic effect when they are stabilized by c-exNDI. In fact, viral replication was here shown to be the step targeted by c-exNDI, which strongly points to the compound G4-based mechanism of action on the HSV-1 G4s. ii) The higher affinity of c-exNDI for HSV-1 G4s *vs* telomeric G4s. These are the most abundant cellular G4s in the infected cell: their lack of optimal recognition by the compound is likely the main reason for the observed low cellular toxicity at antiviral effective doses (Fig. 5).

Impairment of viral replication led to the decrease of viral genes in all phases of the viral life cycle. This effect is shared between c-exNDI and ACV^36^. We have previously shown that the general G4 ligand, BRACO-19, mainly decreased L viral genes^27^. This discrepancy may be due to the lower affinity of BRACO-19 for HSV-1 G4s and lower antiviral activity (IC_50_ in the low micromolar range)^27^ which would result in observable inhibition only of genes temporally more closely influenced by inhibition of viral replication. In contrast, in the case of c-exNDI and ACV, their potent effect on the HSV-1 genome is likely sensed at a more extended level. There is also the fascinating possibility that c-exNDI reacts with additional G4s, such as those in key regulatory IE and E genes; in fact, besides the extended G4 repeats in the terminal and inverted repeats^27^, less extended but stable G4s structures are also distributed throughout the HSV-1 genome and embedded in the promoter and coding sequences of fundamental genes^27^.

The unique mechanism of action of c-exNDI makes it suitable for treatment of HSV-1 strains resistant to current therapies; for instance, the emergence of ACV resistant strains has long created an obstacle for the treatment of HSV-1^38^. Even though c-exNDI has a less wide therapeutic window than ACV, it is active at nanomolar concentrations, a promising feature for its prospect development as anti-herpetic drugs. Since the HSV-1 infection is a common illness associated to immunosuppression, the impact of our data extends to AIDS, cancer and transplanted patients.

## Methods

### Cells and viruses

Vero cells (Sigma-Aldrich, Saint Louis, USA) were grown in DMEM supplemented with 10% FBS and 1X PenStrep antibiotic (Gibco, Life Technologies, Monza, Italy). HSV-1 strain F was a kind gift of B. Roizman (University of Chicago, Illinois, USA). Recombinant HSV-1 expressing VP16-GFP (HSV-1 [V41]) was kindly provided by Peter O’ Hare (Imperial College, London, UK).

### Oligonucleotides and compounds

All oligonucleotides and primers were from Sigma Aldrich (Milan, Italy), Table S1. The G4 ligand c-exNDI was synthesized and provided by Prof. M. Freccero (University of Pavia, Italy). The control compound acyclovir (ACV) was purchased from Sigma Aldrich (Milan, Italy).

### Circular Dichroism

Circular dichroism (CD) experiments were performed using a Chirascan-Plus (Applied Photophysics, Leatherhead, UK) equipped with a Peltier temperature controller using a quartz cell of 5 mm path length. G4 folding oligonucleotides were diluted to a final concentration of 4 μM in the absence or presence (100 mM) of KCl and 10 mM lithium cacodilate buffer. After annealing step (5 min at 95 °C), DNA samples were gradually cooled down and, where specified, c-exNDI was added at a final concentration of 16 μM. Thermal unfolding analyses were recorded from 230 to 320 nm over a temperature range of 20–90 °C (5 °C/min). The reported spectrum of each sample represents the average of 2 scans and is baseline-corrected for buffer contribution. Observed ellipticities were converted to mean residue ellipticity (θ) = deg × cm^2^ × dmol^−1^ (mol ellip). T_m_ values were calculated according to the Van’t Hoff equation, applied for a two-state transition, assuming that the heat capacity of the folded and unfolded states are equal.

### Taq polymerase stop assay

The *Taq* polymerase stop assay was performed as previously described^5^. Briefly, the 5′-end labeled primer (HSV Taq primer, Table S1) was annealed to each template (Table S1) in lithium cacodylate buffer. Where specified, samples were incubated with KCl 10 mM in the presence or absence of c-exNDI (0, 37.5, 75, 150 nM) at rt. Elongation was carried out at 60 °C for 30 min using 2 U of Ampli*Taq* Gold DNA polymerase (Applied Biosystem, Carlsbad, California, USA). All reactions were stopped by ethanol precipitation and primers elongation products were resolved on a 15% polyacrylamide denaturing gel. Gel visualization was performed with phosphoimaging (Typhoon FLA 9000, GE Healthcare, Milan, Italy).

### Mass Spectrometric (MS) competition assay

Oligonucleotides were heat-denatured and folded in 0.8 mM KCl, 120 mM trimethylammonium acetate (TMAA), pH 7.0, and 20% isopropanol (IPA) overnight at 4 °C. Oligonucleotides were diluted to the final concentration of 4 μM and incubated with c-exNDI at DNA:compound 1:1 overnight at 4 °C. Samples were analyzed by direct infusion electrospray ionization (ESI) on a Xevo G2-XS QTof mass spectrometer (Waters, Manchester, UK). The injection was automatically performed by an Agilent 1290 Infinity HPLC (Agilent Technologies, Santa Clara, CA, US) equipped with an auto sampler; the carrying buffer was TMAA 80 mM, 20% IPA. Up to 5 μL samples were typically injected per each analysis. The ESI source settings were the following: electrospray capillary voltage set at 1.8 kV, the source and desolvation temperatures were 45 °C and 65 °C respectively, the sampling cone was set at 65 V. All these parameters ensured minimal fragmentation of the DNAs complexes. The instrument was calibrated using a 2 mg/mL solution of sodium iodide in 50% of IPA. Binding affinities were calculated for each experiment using the reconstructed-ion chromatogram area for each species calculated by MassLynx V4.1. The binding affinity was calculated with the following formula: [BA = (ΣG4b/(ΣG4f + ΣG4b)) × 100], where BA is the binding affinity, G4b is chromatogram area of bound G4 DNA, and G4f is the chromatogram area of free G4 DNA.

### Cytotoxicity assay

Cytotoxicity of c-exNDI in Vero cells was determined by MTT assay. Briefly, serial dilutions of c-exNDI (0.7–12.8 μM) were dispensed to 24 h plated Vero cells (1 × 10^4^ cells/well). After 48 h from treatment, cells were supplemented with freshly diluted 3-(4,5-dimethylthiazol-2-yl)-2,5-diphenyltetrazolium bromide (MTT) (Sigma-Aldrich, Milan, Itlay) solution (5 mg/mL) and incubated for 4 h. After solubilization, absorbance was measured by Sunrise Tecan plate reader (Mannendorf, Switzerland) at 620 nm. The 50% cytotoxic concentration (CC_50_) was determined from the dose-response curve.

### Antiviral assays

The antiviral activity of c-exNDI against HSV-1 (F) was investigated by plaque assay. For virus infection, wild- type (wt) HSV-1 (F) was added to Vero cells at different multiplicities of infection (MOIs) in serum-free medium. After 1 h at 37 °C, the inoculums were replaced with complete medium. After infection, the compound was added at increasing concentrations (0.7–100 nM) in each well. Because a single round of HSV-1 replication takes around 24 h to complete and at this time p.i. the production of virus reaches a plateau^39, 40^, supernatants were collected 24 h.p.i and stored at −80 °C until viral titrations by plaque assay. For plaque reduction assay, Vero cells were seeded in 24-well plate (1 × 10^5^/well) and incubated overnight. Cells were then infected with 250 μl of serially-diluted (10-folds) supernatants for 1 h at 37 °C. After infection, cells were washed with PBS 1X and incubated with 500 μl of DMEM supplemented with 0.6% methylcellulose and 2% FBS. Forty-eight h.p.i., cells were washed with PBS 1X and fixed with formaldehyde 5% in PBS 1X for 10 min at room temperature, then colored with crystal violet 0.8% (in ethanol 50%). Viral plaques were counted using an optical microscope (Zeiss, Jena, Germany).

### Time of addition assay

Time of addition assay (TOA) was performed to establish which is the last step of the viral infection cycle affected by the compound^41^. Vero cells were seeded in 24-well plate (1 × 10^5^ cells/well) and incubated overnight. Cells were then infected with HSV-1 (F) at a MOI of 0.5 (as suggested^41^) and treated every two hours (from 0 to 12 h.p.i) with c-exNDI (100 nM) or ACV (3 μM) as reference drug. To allow estimation of the c-exNDI effects at all viral steps, supernatants were collected 30 h.p.i. and then titrated following plaque assay working protocol as described above.

### Flow cytometry

Vero cells were seeded into 12-wells plates (1 × 10^5^ cells/well) and next day infected with mutant HSV-1 [V41] expressing VP16-GFP using the procedure described above. MOI of 0.3 was used because at higher MOIs the GFP signal reached saturation levels. After infection, Vero cells were incubated for 24 h with complete medium or with complete medium containing c-exNDI (100 nM) or ACV (3 μM). GFP fluorescence was acquired for 30.000 events in each sample using FACS Cytofluorometer BD LSR II (BD Bioscences, New Jersey, USA). Fluorescence acquisitions were analyzed with FlowJo software (Tree Star, Oregon, USA).

### Real-time PCR and qPCR

For total RNA extraction Vero cells were plated (1.8 × 10^5^/well), mock- or HSV-1 (F)-infected (MOI of 3) and treated with c-exNDI 100 nM. At 4 or 24 h.p.i. total RNA was isolated using TRIzol reagent (Life Technologies, Monza, Italy) according to the manufacturer’s instructions and subjected to RNase free DNase I treatment (Ambion Turbo DNA free, Life Technologies, Monza, Italy). Extracted RNA (1 μg) was subjected to reverse transcription by 1.5 U MuLv (Life Technologies, Monza, Italy). Reverse transcription was carried out as follows: 10 min at 25 °C, 60 min at 48 °C and 5 min at 95 °C using the Thermo Cycler Verity 96 (Applied Biosystem, Monza, Italy). Forward/reverse primers were designed within conserved HSV-1 gene sequences using Primer Express 3 (Applied Biosystem, Monza, Italy) Table S2. Real-time PCR was performed using TaqMan chemistry with 5′-[FAM] and 3′-[TAMRA] end labeled probes. Realtime reaction was composed of FAST Master Mix 2X (Applied Biosystem, Monza, Italy), 900 nM of forward/reverse primer mixture, 200 nM of TaqMan probe, sterile water and 3μL of cDNA. Experiments were performed using ABI 7900 HT – FAST RealTime PCR System under the following conditions: 95 °C for 10 min followed by 40 cycles of 30 seconds at 95 °C and 30 seconds at 58 °C, 1 minute at 72 °C. Messenger RNA transcription levels were standardized against the housekeeping gene β-actin. Non- treated retrotranscribed HSV-1 RNA was used as mRNA expressed control. Each sample was analyzed in duplicate.

For quantitative PCR (qPCR) cells were plated (1.8 × 10^5^/well), mock- or HSV-1 (F)-infected (MOI of 1) and treated with c-exNDI at 100 nM. At 4 or 24 h.p.i. cells were collected and total DNA was extracted using NucleoSpin Tissue (Macherey- Nagel, Duren, Germany) according to the manufacturer’s instruction. Isolated intracellular DNA was analyzed by realtime PCR using TaqMan chemistry with 5′-[FAM] and 3′-[TAMRA]-end labeled probes (Table S2) in a final volume of 25 μL. qPCR reaction was composed of FAST Master Mix 2X (Applied Biosystem, Monza, Italy), 500 nM of forward/reverse primer mixture (gene US1, Table S2), 200 nM of TaqMan probe, sterile water and 10 μL of isolated DNA. Experiments were performed using ABI 7900 HT – FAST RealTime PCR System under the following conditions: 95 °C for 5 min followed by 45 cycles of 5 seconds at 95 °C and 10 seconds at 60 °C. Each sample was analyzed in duplicate.

## Electronic supplementary material

Supplementary info
