# Supplementary material for: A core extended naphtalene diimide G-quadruplex ligand potently inhibits herpes simplex virus 1 replication
Source: Sci Rep. 2017 May 24;7:2341. doi: 10.1038/s41598-017-02667-3 (PMC5443766; doi:10.1038/s41598-017-02667-3)

A core extended naphtalene diimide G-quadruplex ligand potently inhibits herpes simplex virus 1 replication

Sara Callegaro[1], Rosalba Perrone[1], Matteo Scalabrin[1], Filippo Doria[2], Giorgio Palù, Sara N. Richter[1]

[1]Department of Molecular Medicine, University of Padua, via Gabelli 63, 35121 Padua, Italy.

[2]Department of Chemistry, University of Pavia, V.le Taramelli 10, 27100 Pavia, Italy.

Table S1. Oligonucleotide sequences used in this study for circular dichroism (CD), mass spectrometry (MS) competition assay and *Taq* polymerase stop assay.

Technique	Oligo Name	Oligo sequence (5'- 3')
CD/MS	*gp054a*	GGGGTTGGGGCTGGGGTTGGGG
	un2	GGGGGCGAGGGGCGGGAGGGGGCGAGGGG
	un3	GGGAGGAGCGGGGGGAGGAGCGGG
	hTel21	GGGTTAGGGTTAGGGTTAGGG
	c-myc	TGGGGAGGGTGGGGAGGGTGGGGAAGG
	c-kit2	CGGGCGGGCGCGAGGGAGGGG
	LTR-III	TGGGAGGCGTGGCCTGGGCGGGACTGGGGT
Taq Polymerase stop assay	HSV Taq primer	GGCAAAAAGCAGCTGCTTATATGCAG
	HSV Taq no G4 cnt	TTGTCGTTAAAGTCTGACTGCGAGCTCTCA GATCCTGCATATAAGCAGCTGCTTTTTGCC
	gp054a	TTTTTGGGGTTGGGGCTGGGGTTGGGGTTTTTCTG CATATAAGCAGCTGCTTTTTGCC
	un2	TTTTTGGGGGCGAGGGGCGGGAGGGGGCGAGGGG TTTTTCTGCATATAAGCAGCTGCTTTTTGCC
	un3	TTTTTGGGAGGAGCGGGGGGAGGAGCGGGTTTTT CTGCATATAAGCAGCTGCTTTTTGCC

Table S2. Primer and probe sequences to perform TaqMan real-time PCR and qPCR. Fw stands for "forward primer, Rv for "reverse primer", Pr for "probe".

Gene name	Gene class	Primer/ Probe	Sequence (5'- 3')	Viral protein	Amplicon lenght (bp)
**US1**	α	Fw<br>Rev<br>Pr	GGCCCGGAGTGTGATCTTAG<br>GGTGGCATCGGAGATTTCAT<br>[FAM]AGATTCATCTCAGCGCGACAAGCGA[TAMRA]	**ICP22**	70
**US12**	α	Fw<br>Rev<br>Pr	CAACGGGTTACCGGATTACG<br>TTGGGTGTGGCACATCGA<br>[FAM]ACTGTCGGTCACGGTCCCGCC[TAMRA]	**ICP47**	68
**UL30**	β	Fw<br>Rev<br>Pr	TTCGACTTTGCCAGCCTGTA<br>CAGGGAGAGCGTGCTGAAG<br>[FAM]AGCATCATCCAGGCCCACAACCTG[TAMRA]	**DNA polymerase**	69
**UL36**	γ	Fw<br>Rev<br>Pr	AGGGAGGATGCCCACGAA<br>TCCGCGTCTTCCACAAATC<br>[FAM]ACACCCCAGACAAGGAGCTGCCC[TAMRA]	**Ubiquitin specific protease**	68
**β-actin**	HK	Fw<br>Rev<br>Pr	TCACTGAGCGCGGCTACA<br>CCTTAATGTCACGCACGATTTC<br>[FAM]TCACCACCACGGCCGAGCG[TAMRA]	**β-actin**	69

Fig. S1. Effect of c-exNDI on HSV-1 DNA replication. Infected cells were treated with c-exNDI (100 nM); 4 or 24 h.p.i., total DNA was isolated and quantified by qPCR (US1 gene). Each sample was analysed in duplicate.

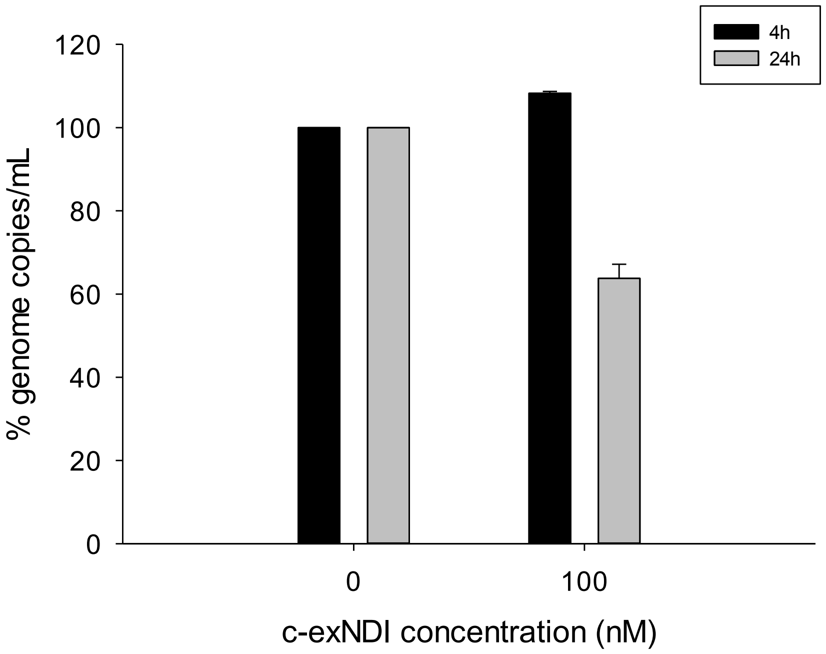

Supplement: Supplementary file 1 — Supplementary info [file 41598_2017_2667_MOESM1_ESM.pdf]
